# Health-related quality of life in patients receiving first-line eribulin mesylate with or without trastuzumab for locally recurrent or metastatic breast cancer

**DOI:** 10.1186/s12885-019-5674-5

**Published:** 2019-06-13

**Authors:** Lee Schwartzberg, Kristi McIntyre, Sharon Wilks, Shannon Puhalla, Joyce O’Shaughnessy, Erhan Berrak, Yaohua He, Linda Vahdat

**Affiliations:** 1 West Cancer Center, 7945 Wolf River Blvd, Germantown, TN 38138, TN 38120 USA; 2 Texas Oncology-Dallas Presbyterian Hospital, US Oncology, Dallas, TX USA; 3 US Oncology-Cancer Care Centers of South Texas, San Antonio, TX USA; 4 University of Pittsburgh Medical Center, Pittsburgh, PA USA; 5 Baylor University Medical Center, Texas Oncology, US Oncology, Dallas, TX USA; 6 Eisai Inc. (former employees), Woodcliff Lake, NJ USA; 7 Weill Cornell Medical College, New York, NY USA

**Keywords:** Eribulin, First-line therapy, Health-related quality of life, Metastatic breast cancer, Trastuzumab

## Abstract

**Background:**

Eribulin mesylate is a nontaxane microtubule dynamics inhibitor approved for second-line (European Union) or third-line (United States) treatment of metastatic breast cancer. Two phase 2 single trials, evaluating first-line eribulin as monotherapy (Study 206; NCT01268150) or in combination with trastuzumab (Study 208; NCT01269346) in locally recurrent or metastatic breast cancer, demonstrated objective response rates of 28.6 and 71.2%, respectively. Median progression-free survival was 6.8 and 11.6 months, respectively. Tolerability profiles were similar to those from previous studies. This secondary analysis was conducted to assess health-related quality of life (HRQoL) in both phase 2 trials.

**Methods:**

Patients received eribulin mesylate 1.4 mg/m^2^ intravenously on days 1 and 8 of each 21-day cycle. Patients in Study 208 also received intravenous trastuzumab on day 1 of each cycle (8 mg/kg in cycle 1, then 6 mg/kg). HRQoL was assessed by the European Organization for Research and Treatment of Cancer Quality-of-Life (QLQ-C30) assessment tool and the Quality-of-Life Questionnaire for Breast Cancer (QLQ-BR23) at baseline and cycles 2, 4, and 6. Results for clinically meaningful changes were based on previously published minimum important differences.

**Results:**

Of the 108 patients (56 in Study 206 and 52 in Study 208) treated, 57 and 87%, respectively, completed 6 cycles. Completion rates for both questionnaires were 94 and 98%, respectively, at cycle 6. Most patients had stable/improved HRQoL scores with some exceptions; for example, more patients experienced a worsening in cognitive functioning and systemic therapy side effects than experienced improvement. Mean QLQ-C30 symptom scores correlated with corresponding adverse event rates for nausea/vomiting, dyspnea, appetite loss, constipation, and diarrhea in Study 206 and for fatigue, nausea/vomiting, pain, dyspnea, insomnia, constipation, and diarrhea in Study 208.

**Conclusions:**

First-line eribulin ± trastuzumab therapy did not lead to deterioration of overall HRQoL in most patients, with more than 60% of patients having stable/improved global health status/quality-of-life scores. Eribulin has been demonstrated to be comparable with other chemotherapy agents with an acceptable safety profile. Therefore, further evaluation is warranted to determine whether eribulin ± trastuzumab therapy may be a potential option for first-line treatment in some patients with metastatic breast cancer who were recently treated in the neoadjuvant setting.

**Trial registration:**

NCT01268150 (December 29, 2010), NCT01269346 (January 4, 2011)

**Electronic supplementary material:**

The online version of this article (10.1186/s12885-019-5674-5) contains supplementary material, which is available to authorized users.

## Background

Advanced or metastatic breast cancer (MBC) typically has a negative impact on health-related quality of life (HRQoL). Patients may experience pain and other physical symptoms due to increasing tumor burden and metastases [1], and some may develop depression or anxiety related to the diagnosis, treatments, concerns about the future, and body image [2, 3]. In addition, adverse events (AEs) related to MBC treatments can negatively affect HRQoL [2].

Because available treatments for MBC are palliative rather than curative, preserving HRQoL is an important aspect of treatment selection, along with measures such as reducing tumor burden and prolonging progression-free survival (PFS) [2, 4]. Thus, evaluation of HRQoL outcomes is particularly relevant in studies of MBC therapies.

Eribulin mesylate, a nontaxane microtubule dynamics inhibitor [5], has demonstrated an overall survival benefit relative to other commonly used agents in patients who have received ≥2 prior MBC therapies, including an anthracycline and taxane [6]. In a randomized, open-label, phase 3 study that compared eribulin with capecitabine for MBC treatment in women who had previously been treated with anthracyclines and taxanes [7], HRQoL was assessed using the European Organization for Research and Treatment of Cancer (EORTC) quality-of-life (QoL) (QLQ-C30) assessment tool [8] and the Quality-of-Life Questionnaire for Breast Cancer (QLQ-BR23) [9]. Scores for global health status (GHS)/QoL improved in both arms, with greater improvements in mean score for eribulin (13.5) than for capecitabine therapy (8.3). No significant differences were observed between the 2 groups, according to a linear mixed model (estimated treatment effect − 0.068, *P* = 0.958) and pattern-mixture model (estimated treatment effect 0.082, *P* = 0.949) [7].

Trastuzumab is a human epidermal growth factor receptor 2 (HER2)-targeted humanized monoclonal antibody; previous clinical data have shown that the addition of trastuzumab to chemotherapy significantly improved overall survival, PFS, and disease-free survival relative to the use of chemotherapy alone in patients with HER2-positive (HER2+) MBC [10, 11]. Studies of trastuzumab, as monotherapy or in combination with chemotherapy, have also demonstrated a beneficial effect on HRQoL in patients with HER2+ MBC [12].

Recently, 2 phase 2 trials evaluating the use of eribulin ± trastuzumab as first-line therapy in locally recurrent or MBC were conducted. Eribulin monotherapy in patients with HER2-negative (HER2-) breast cancer (Study 206) [13] demonstrated an objective response rate (ORR) of 28.6%, overall clinical benefit rate (CBR) of 51.8%, and PFS of 6.8 months. Combination therapy of eribulin plus trastuzumab as first-line therapy in patients with HER2+ breast cancer (Study 208) [14] showed an ORR of 71.2%, CBR of 84.6%, and PFS of 11.6 months. The present secondary analysis was conducted to assess the HRQoL results from the first 6 treatment cycles of these phase 2 trials of eribulin ± trastuzumab as first-line therapy for locally recurrent or MBC.

## Methods

### Study design

**Fig. 1:**
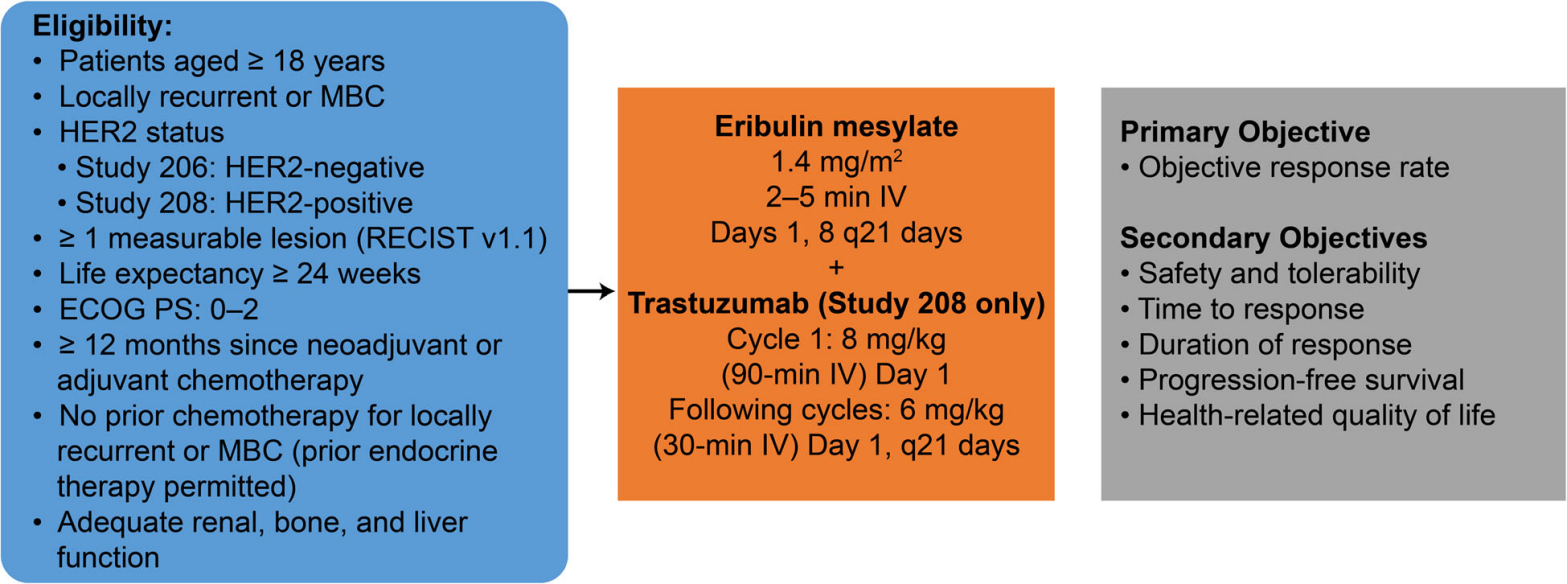
Studies 206 and 208 designs

These phase 2 studies were originally conducted to explore the antitumor activity and safety of eribulin monotherapy as first-line therapy in patients with locally recurrent or metastatic HER2- breast cancer (Study 206), and in combination with trastuzumab for patients with HER2+ breast cancer (Study 208). After completing 6  cycles of therapy in the treatment phase, patients were eligible to enter an extension phase in which they continued treatment until disease progression. Full details of the study designs have been published [13, 14]. Patient eligibility, study treatments, and study end points are shown in Fig. 1. Both studies were conducted in accordance with the Declaration of Helsinki (2008), and the protocol and informed consent forms were submitted for approval to institutional review boards. All patients provided written informed consent.

### HRQoL assessments

HRQoL was assessed using the EORTC QLQ-C30 and QLQ-BR23 at baseline and day 1 of every other cycle (eg, C2D1, C4D1, C6D1) during both the treatment and extension phases. Designed with multi- and single-item scales, the QLQ-C30 is a 30-item questionnaire assessing function and symptoms that impact QoL in patients with cancer [8]. It was developed as an integrated measurement system for international clinical trials and incorporates 5 functional scales (physical, role, cognitive, emotional, and social), 3 symptom scales (fatigue, pain, and nausea and vomiting), and 1 global health and QoL scale. Several single-item measures assess common cancer symptoms (eg, dyspnea, loss of appetite, sleep disturbance, constipation, and diarrhea) and the perceived financial impact of cancer and its treatments [8]. The QLQ-BR23 is a breast cancer-specific 23-item questionnaire that was designed to be used in conjunction with the QLQ-C30 to evaluate HRQoL in international clinical trials. It incorporates 2 functional scales (body image and sexuality), 3 symptom scales (arm symptoms, breast symptoms, and systemic-therapy side effects), and single items on sexual enjoyment, hair loss, and future perspective [9].

### Statistical analysis

Completion rates, scores, and summary statistics for the QLQ-C30 and QLQ-BR23 questionnaires were summarized at each assessment time point. An HRQoL form was considered complete if the patient answered at least 1 question. Clinically meaningful changes from baseline for each of the QLQ-C30 and QLQ-BR23 domains and single items were summarized at each time point. Percentages of patients in the “improved,” “stable,” or “worsened” categories were calculated based on a minimum important difference of ±10 points. For global health status and functional scales (QLQ-C30 and QLQ-BR23), “improved” scores were defined as a ≥ 10-point increase from baseline and “worsened” was defined as a ≥ 10-point decrease from baseline. For Symptom scales/items (QLQ-C30 and QLQ-BR23), “improved” scores were defined as decreases from baseline ≥10 points and “worsened” was defined as a ≥ 10-point increase from baseline. The value of 10 was selected to define a clinically meaningful difference based on previous randomized clinical trials where it was most common to assume that 10 points was clinically significant [15, 16]. These data were analyzed for the overall population. To assess the clinical relevance of each HRQoL symptom scale, the Spearman’s rank correlation coefficient between individual HRQoL symptom scores and their corresponding AE rates (number of events divided by duration from the first date of study medication to the date of discontinuation from treatment + 30 days) was calculated.

## Results

### Patients

Study 206 enrolled 56 patients and Study 208 enrolled 52 patients, and 32 (57.1%) and 45 (86.5%) patients completed 6  cycles of therapy, respectively. Reasons for discontinuation included disease progression (*n* = 18 and *n* = 3, respectively), AEs (*n* = 3 in both studies), and patient choice (*n* = 3 and *n* = 1, respectively). Most patients (76%) were Caucasian with a median age of 56 and 60 years, respectively. The median time from diagnosis was 2.7 and 2.1 years, respectively. The majority of patients had disease that was estrogen receptor- or progesterone receptor-positive, and with the most common metastatic sites were bone, liver, and lung (Table 1).

**Table 1:** Baseline demographics and disease characteristics

Characteristic	Study 206 (*N* = 56)	Study 208 (*N* = 52)
Age, years		
Median (range)	56 (31, 85)	60 (31, 81)
Mean (SD)	57 (11)	59 (11)
Female, n (%)	56 (100)	51 (98)
Race, n (%)		
Caucasian	42 (75)	40 (77)
Black or African American	12 (21)	11 (21)
Asian	1 (2)	1 (2)
Other^a^	1 (2)	0
Ethnicity, n (%)		
Hispanic or Latino	3 (5)	5 (10)
Not Hispanic or Latino	53 (95)	47 (90)
Mean time from original diagnosis of breast cancer, years (SD)	5 (6)	3 (3)
Diagnosis of malignant disease, n (%)		
Ductal adenocarcinoma	42 (75)	46 (89)
Lobular adenocarcinoma	3 (5)	3 (6)
Other	11 (20)	3 (6)
ER/PR/HER2 status, n (%)		
ER+ or PR+	44 (79)	36 (69)
ER- and PR-	12 (21)	15 (29)
HER2+	0	52 (100)
HER2-	56 (100)	0
Site of metastasis, n (%)		
Bone	37 (66)	19 (37)
Liver	25 (45)	25 (48)
Lung	22 (39)	24 (46)
Skin	5 (9)	3 (6)
Other	35 (63)	33 (64)
Lymph node involvement only	3 (5)	4 (8)

*ER* estrogen receptor, *HER* human epidermal growth factor receptor, *PR* progesterone receptor, *SD* standard deviation

^a^Other race: Non-Caucasian Hispanic

### Efficacy

The ORR in Study 206 was 28.6% (95% CI: 17.30, 42.21), and in Study 208, it was 71.2% (95% CI: 56.92, 82.87). Results for secondary efficacy outcomes included median PFS of 6.8 months (95% CI: 4.44, 7.59) and 11.6 months (95% CI: 9.13, 13.93); median time to response of 1.4  months (95% CI: 1.22, 2.66) and 1.3  months (95% CI: 1.22, 1.38); median duration of response of 5.8 months (95% CI: 4.67, 10.55) and 11.1 months (95% CI: 6.70, 17.77); and CBR (CR + PR + durable stable disease) of 51.8% (95% CI: 38.03, 65.34) and 84.6% (95% CI: 71.92, 93.12), for Studies 206 and 208, respectively. Additional efficacy results were previously published [13, 14].

### HRQoL scores

The completion rate (ie, percentage of patients who completed at least 1 item) for the QLQ-C30 and QLQ-BR23 was 94% (30/32) in Study 206 and 98% (44/45) in Study 208 at cycle 6 among patients who remained in the studies.

### Clinically meaningful changes

**Fig. 2:**
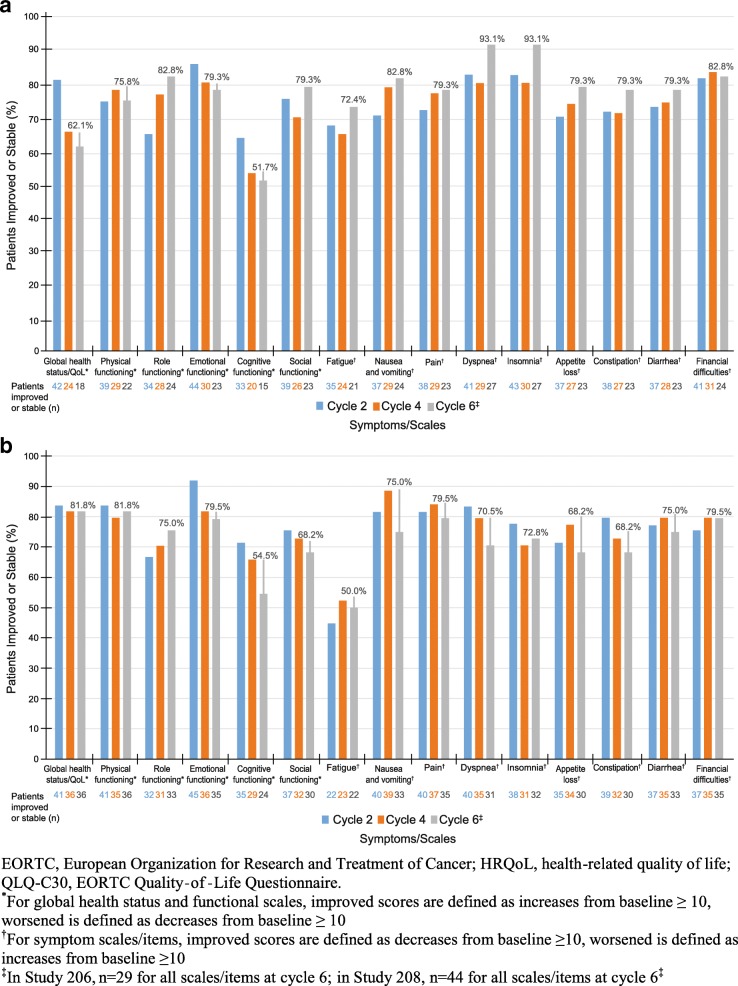
Proportion of patients who maintained/improved HRQoL – EORTC QLQ-C30 in Study 206 (**a**) and Study 208 (**b**), ITT population

In both studies, most patients had improved HRQoL scores for QLQ-C30 at cycle 6 (Table 2). At cycle 6, more patients in Study 206 had an improvement (increases from baseline ≥10 for GHS and functional scales; decreases from baseline ≥10 for symptom scales) from baseline for physical functioning, role functioning, emotional functioning, social functioning, fatigue, nausea and vomiting, pain, dyspnea, insomnia, and appetite loss than had a worsening. More patients experienced a worsening in GHS/QoL, cognitive functioning, constipation, diarrhea, and financial difficulties than had an improvement. In Study 208, more patients experienced an improvement from baseline for GHS/QoL, physical functioning, role functioning, emotional functioning, pain, insomnia, and financial difficulties than experienced a worsening. More patients experienced a worsening for cognitive functioning, social functioning, fatigue, nausea and vomiting, dyspnea, appetite loss, constipation, and diarrhea than experienced an improvement. Figure 2a and 2b display the proportion of patients whose QLQ-C30 scores were stable/improved at cycles 2, 4, and 6. Baseline QoL scores are summarized in Additional file 1: Table S1.

**Table 2:** Percentage of patients with improved, stable, or worsened QLQ-C30 scores from baseline at cycle 6

Symptom/Scale	Study 206				Study 208			
	n	Improved (%)	Stable (%)	Worsened (%)	n	Improved (%)	Stable (%)	Worsened (%)
GHS/QoL	29	6.9	55.2	37.9	44	29.5	52.3	18.2
Physical functioning	29	31.0	44.8	24.1	44	22.7	59.1	18.2
Role functioning	29	48.3	34.5	17.2	44	29.5	45.5	25.0
Emotional functioning	29	31.0	48.3	20.7	44	38.6	40.9	20.5
Cognitive functioning	29	6.9	44.8	48.3	44	13.6	40.9	45.5
Social functioning	29	34.5	44.8	20.7	44	27.3	40.9	31.8
Fatigue	29	55.2	17.2	27.6	44	31.8	18.2	50.0
Nausea and vomiting	29	27.6	55.2	17.2	44	20.5	54.5	25.0
Pain	29	51.7	27.6	20.7	44	47.7	31.8	20.5
Dyspnea	29	31.0	62.1	6.9	44	27.3	43.2	29.5
Insomnia	29	41.4	51.7	6.9	44	36.4	36.4	27.3
Appetite loss	29	24.1	55.2	20.7	44	22.7	45.5	31.8
Constipation	29	17.2	62.1	20.7	44	25.0	43.2	31.8
Diarrhea	29	6.9	72.4	20.7	44	9.1	65.9	25.0
Financial difficulties	29	13.8	69.0	17.2	44	29.5	50.0	20.5

*GHS* global health status, *QoL* quality of life

For global health status and functional scales, improved scores are defined as increases from baseline ≥10; worsened is defined as decreases from baseline ≥10. For symptom scales/items, improved scores are defined as decreases from baseline ≥10; worsened is defined as increases from baseline ≥10

**Fig. 3:**
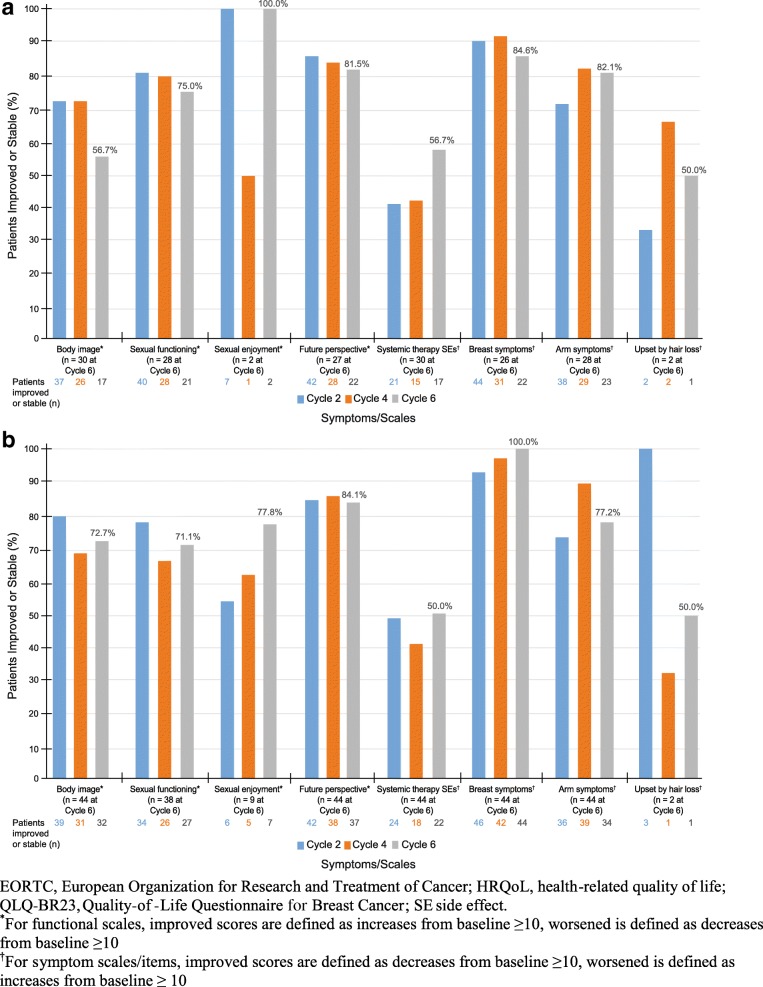
Proportion of patients who maintained/improved HRQoL—EORTC QLQ-BR23 in Study 206 (**a**) and Study 208 (**b**), ITT population

Most patients had stable HRQoL scores for QLQ-BR23 at cycle 6 (Table 3). At cycle 6, more patients in Study 206 had an improvement (increases from baseline ≥10 for GHS and functional scales; decreases from baseline ≥10 for symptom scales) for future perspective, breast symptoms, and arm symptoms than had a worsening, respectively. More patients experienced a worsening in body image, sexual functioning, systemic therapy side effects, and “upset by hair loss” scores than had improvement. In Study 208, more patients experienced an improvement from baseline for future perspective, breast symptoms, and arm symptoms than had a worsening, respectively. More patients experienced a worsening in body image, sexual functioning, sexual enjoyment, systemic therapy side effects, and “upset by hair loss” than had improvement. Figures 2 and 3 display the proportion of patients whose QLQ-BR23 scores were stable/improved at cycles 2, 4, and 6.

**Table 3:** Percentage of patients with improved, stable, or worsened QLQ-BR23 scores from baseline at cycle 6

Symptom/Scale	Study 206				Study 208			
	n	Improved (%)	Stable (%)	Worsened (%)	n	Improved (%)	Stable (%)	Worsened (%)
Body image^a^	30	6.7	50.0	43.3	44	18.2	54.5	27.3
Sexual functioning^a^	28	10.7	64.3	25.0	38	7.9	63.2	28.9
Sexual enjoyment^a^	2	0	100	0	9	11.1	66.7	22.2
Future perspective^a^	27	22.2	59.3	18.5	44	40.9	43.2	15.9
Systemic therapy SEs^b^	30	3.3	53.3	43.3	44	4.5	45.5	50.0
Breast symptoms^b^	26	23.1	61.5	15.4	44	31.8	68.2	0
Arm symptoms^b^	28	46.4	35.7	17.9	44	47.7	29.5	22.7
Upset by hair loss^b^	2	0	50.0	50.0	2	0	50.0	50.0

*QLQ-BR23* Quality-of-Life Questionnaire for Breast Cancer, *SE* side effect

^a^For global health status and functional scales, improved scores are defined as increases from baseline ≥10; worsened is defined as decreases from baseline ≥10

^b^For symptom scales/items, Improved scores are defined as decreases from baseline ≥10; worsened is defined as increases from baseline ≥10

For both studies, changes in outcomes were also assessed in relation to treatment response, but because of the small number of patients following stratification by response, the data were insufficiently robust enough to allow for meaningful interpretations. However, the data were generally consistent with the overall observation that scores remained stable or improved for a majority of patients during treatment.

### Correlation with adverse events

The most common treatment-related AEs (TRAEs) of any grade (incidence ≥25%) in Study 206 [13] were alopecia (83.9%), neutropenia (71.4%), fatigue (60.7%), nausea (48.2%), peripheral neuropathy (44.6%), anemia (35.7%), leukopenia (33.9%), constipation (26.8%), and diarrhea (25.0%). Grade 3 or 4 TRAEs occurred in 36 patients (64.3%), with the most common being neutropenia (50.0%), peripheral neuropathy (19.6%), and leukopenia (21.4%). In Study 208 [14], common TRAEs of any grade included alopecia (86.5%), fatigue (57.7%), peripheral neuropathy (59.6%), neutropenia (59.6%), nausea (36.5%), and anemia (25%). Grade 3 or 4 TRAEs occurred in 31 patients (59.6%), with the most common being neutropenia (38.5%) and peripheral neuropathy (21.2%). Mean symptom scales were significantly correlated with corresponding AE rates (nausea and vomiting, dyspnea, appetite loss, constipation, and diarrhea for Study 206; fatigue, nausea/vomiting, pain, dyspnea, insomnia, constipation, and diarrhea for Study 208. The only QLQ-BR23 item with a corresponding AE was “upset by hair loss,” which was not significantly correlated in either study (Table 4).

**Table 4:** Correlation between HRQoL symptoms and selected adverse events

Symptom/Scale	Study 206 (*N* = 56)		Study 208 (*N* = 52)	
	Spearman Correlation Coefficient (95% CI)	*P* Value	Spearman Correlation Coefficient (95% CI)	*P* Value
QLQ-C30				
Fatigue	0.14 (−0.13, 0.39)	0.3059	0.31 (0.03, 0.53)	0.0260
Nausea and vomiting	0.33 (0.07, 0.55)	0.0117	0.50 (0.26, 0.68)	0.0001
Pain	0.13 (−0.14, 0.37)	0.3586	0.41 (0.15, 0.61)	0.0025
Dyspnea	0.31 (0.05, 0.53)	0.0201	0.49 (0.25, 0.67)	0.0002
Insomnia	0.24 (−0.03, 0.47)	0.0809	0.35 (0.08, 0.57)	0.0104
Appetite loss	0.35 (0.09, 0.56)	0.0080	0.23 (−0.05, 0.47)	0.1055
Constipation	0.31 (0.05, 0.53)	0.0191	0.30 (0.03, 0.53)	0.0283
Diarrhea	0.54 (0.32, 0.70)	< 0.0001	0.40 (0.14, 0.60)	0.0032
QLQ-BR23				
Upset by hair loss^a^	0.16 (−0.11, 0.41)	0.2345	0.04 (−0.24, 0.31)	0.8026

*CI* confidence interval, *HRQoL* health-related quality of life, *QLQ-C30* Quality-of-Life Questionnaire for Patients with Cancer, *QLQ-BR23* Quality-of-Life Questionnaire for Breast Cancer

^a^*n* ≤ 10 at each visit

## Discussion

In these phase 2 studies, eribulin mesylate ± trastuzumab was evaluated as first-line therapy for locally recurrent or MBC. After 6  cycles, treatment did not lead to deterioration of overall HRQoL in most patients, with more than 60% of patients having stable/improved EORTC QLQ-C30 GHS/QoL scores. Minor differences were observed between the 2 trials, mainly in overall QOL with preservation over time in Study 208 and some deterioration in Study 206. Patients with stable/improved scores for the other items ranged from 51.7 to 93.2% in the QLQ-C30 and 50.0 to 100.0% in the QLQ-BR23 questionnaire. Most patients in both studies experienced improved pain (51.7 and 47.7%) and arm symptom scores (46.4 and 47.7%) with first-line eribulin, while most also had worsened cognitive functioning (48.3 and 45.5%). In Study 208, most patients also experienced worsened systemic therapy side effects (50%).

Significant correlations were observed between some of the most commonly reported AEs (fatigue, nausea and vomiting, pain, dyspnea, insomnia, constipation, and diarrhea) and corresponding HRQoL symptom items. Alopecia was a notable exception, due to a very limited number of responses for that item (*n* = 2 at cycle 6 for both studies). These correlations demonstrate consistency between investigator-reported adverse events and patient-reported outcomes.

It should be noted that as patients discontinued from the study, fewer patients were available for certain HRQoL assessments. For the scale of “upset by hair loss,” fewer than 10 patients for each timepoint and each visit were available. For the scale of “sexual enjoyment,” fewer than 10 patients for each time and each visit were available, with the exception of cycle 2 in which there were 11 patients. Similarly, the relatively small subgroups of responders and nonresponders hinder the ability to draw any conclusions regarding differences in HRQoL outcomes as a function of efficacy, or to evaluate whether HRQoL was a significant predictor of response as has been shown in other studies [17–20]. A larger patient sample would help to better demonstrate the effects of eribulin ± trastuzumab on these and other aspects of HRQoL.

While combination chemotherapy for MBC provides higher ORR, longer times to progression, and a modest survival benefit, it is also associated with increased toxicity [21]. A systematic review published in 2005 [21] that compared the use of single-agent chemotherapy with combination chemotherapy for MBC analyzed 9 trials that evaluated QoL, only 4 of which found statistically significant differences. Of the 4 trials, 2 (Heidemann et al. [22] and Joensuu et al. [23]) found improved QoL with single-agent therapy. Heidemann et al. used Brunner’s score to assess QoL [24] and found that single-agent therapy with mitoxantrone resulted in improved QoL scores for hair loss, nausea, and vomiting compared with combination chemotherapy. Joensuu et al. utilized the Rotterdam Symptom Checklist [25] and found improved QoL scores for physical distress and nausea with epirubicin therapy compared with combination therapy. The remaining 2 studies (Nabholtz et al. [26] and Simes et al. [27]) reported mixed results. Using the EORTC QLQ-C30 questionnaire [21], Nabholtz et al. [26] reported that patients who received single-agent docetaxel experienced improved QoL scores for nausea/vomiting and loss of appetite while patients who received mitomycin and vincristine experienced improved QoL scores for role functioning and social functioning. Simes et al. [27] reported improved QoL scores using the Spitzer QoL index [28] during the first 3 months for pain, mood, and nausea and vomiting in the combination therapy group, but decreased QoL scores for hair loss compared with mitoxantrone monotherapy [21]. A subsequent systematic review and meta-analysis of combination versus sequential single-agent therapies reported no significant difference in QoL, although only 3 of the 12 included trials reported on QoL [29].

The results from Study 208 are generally consistent with those from a study of trastuzumab in combination with chemotherapy for HER2+ MBC, which also utilized the EORTC-QLQ-30 [30]. In that study, patients treated with trastuzumab plus anthracycline/cyclophosphamide or paclitaxel chemotherapy (*n* = 208) and patients treated with chemotherapy alone (*n* = 192) had an initial worsening in fatigue, physical functioning, role functioning, social functioning, and global QoL at week 8 during treatment; patients in the trastuzumab plus chemotherapy group generally showed greater improvements in these domains following cessation of chemotherapy at week 20, with a statistically significantly greater improvement in fatigue at 32 weeks (*P* < 0.05). In addition, the percentage of patients with improvement ≥10 points in global QoL was significantly greater among patients who received trastuzumab plus chemotherapy (51%) compared with the percentage among patients who received chemotherapy alone (36%; *P* < 0.05 after Bonferroni correction) [30]. In the CLEOPATRA study, HRQoL was evaluated using the Trial Outcome Index-Physical/Functional/Breast (TOI-PFB) of the Functional Assessment of Cancer Therapy-Breast (FACT-B) [31]. In that study, patients (*n* = 806) treated with trastuzumab plus docetaxel (with or without pertuzumab) showed an initial decline in mean QoL scores during the first 6  cycles, which then returned to baseline; the median time to deterioration was approximately 18 weeks [31]. The timing of the decline in TOI-PFB scores relative to the timing of docetaxel discontinuation suggested that AEs associated with docetaxel may have been a factor [31]. In the AVEREL study, which assessed HRQoL using the FACT-B scale, mean scores among patients treated with docetaxel and trastuzumab (*n* = 174) decreased throughout the first 5 cycles, then increased at cycle 11, showing slight improvement over baseline; mean scores among patients who also received bevacizumab (*n* = 185) remained relatively stable through cycle 5, then increased at cycle 11 [32]. In a study that compared trastuzumab plus docetaxel (*n* = 70) with trastuzumab emtansine (*n* = 67), the median time to decrease of 5 or more points on the FACT-B TOI-PFB was 3.5 months in the trastuzumab plus docetaxel group and was 7.5 months in the trastuzumab–emtansine group (*P* = 0.022) [33].

## Conclusions

The results of this secondary analysis of two phase 2 trials demonstrate that most patients experience stable or improved HRQoL scores when receiving eribulin mesylate ± trastuzumab as first-line therapy for locally recurrent or MBC. However, there were some exceptions; more patients experienced a worsening in cognitive functioning and systemic therapy side effects than experienced an improvement.

Efficacy data from these phase 2 trials also demonstrate that response rates with eribulin monotherapy are in line with what has been observed in trials of single-agent anthracyclines or taxanes in a similar treatment setting, and combination therapy with trastuzumab yields similar results as in previous studies [30–33]. Tolerability profiles and maintenance of symptom control were favorable and consistent with previous studies [13, 14]. Therefore, further evaluation is warranted to determine whether eribulin ± trastuzumab therapy may be a potential option for first-line treatment for some patients with metastatic breast cancer who were recently treated in the neoadjuvant setting.

## Additional file

Additional file 1: **Table S1.** Summary of QLQ-C30 and QLQ-BR23 Baseline Scores. (DOCX 16 kb)

## Abbreviations

AE: Adverse event
CBR: Clinical benefit rate
CR: Complete response
EORTC: European Organization for Research and Treatment of Cancer
FACT-B: Functional Assessment of Cancer Therapy-Breast
GHS: Global health status
HER2: Human epidermal growth factor receptor 2
HRQoL: Health-related quality of life
MBC: Metastatic breast cancer
ORR: Objective response rate
PFS: Progression-free survival
PR: Partial response
QLQ-BR23: Quality-of-Life Questionnaire for Breast Cancer
QLQ-C30: Quality-of-Life Questionnaire assessment tool
QoL: Quality of life
TOI-PFB: Trial Outcome Index-Physical/Functional/Breast
TRAE: Treatment-related adverse event

## References

[1] Irvin W, Muss HB, Mayer DK (2011). Symptom management in metastatic breast cancer. Oncologist..

[2] Twelves C, Gradishar WJ, O’Shaughnessy JA, Bramsen B, Lurie RH (2014). Clinical roundtable monograph: effective management of quality of life in metastatic breast cancer. Clin Adv Hematol Oncol.

[3] Grabsch B, Clarke DM, Love A, McKenzie DP, Snyder RD, Bloch S (2006). Psychological morbidity and quality of life in women with advanced breast cancer: a cross-sectional survey. Palliat Support Care.

[4] National Comprehensive Cancer Network (2014). NCCN clinical practice guidelines in oncology (NCCN guidelines)®: breast Cancer version 3.2014.

[5] Eisai Inc (2014). Halaven® (eribulin mesylate) injection [prescribing information].

[6] Cortes J, O’Shaughnessy J, Loesch D, Blum JL, Vahdat LT, Petrakova K (2011). Eribulin monotherapy versus treatment of physician's choice in patients with metastatic breast cancer (EMBRACE): a phase 3 open-label randomised study. Lancet..

[7] Kaufman PA, Awada A, Twelves C, Yelle L, Perez EA, Velikova G (2015). Phase III, open-label, randomized study of eribulin mesylate versus capecitabine in patients with locally advanced or metastatic breast cancer previously treated with an anthracyclines and a taxane. J Clin Oncol.

[8] Aaronson NK, Ahmedzai S, Bergman B, Bullinger M, Cull A, Duez NJ (1993). The European Organization for Research and Treatment of Cancer QLQ-C30: a quality-of-life instrument for use in international clinical trials in oncology. J Natl Cancer Inst.

[9] Sprangers MA, Groenvold M, Arraras JI, Franklin J, te VA, Muller M (1996). The European Organization for Research and Treatment of Cancer breast cancer-specific quality-of-life questionnaire module: first results from a three-country field study. J Clin Oncol.

[10] Slamon DJ, Leyland-Jones B, Shak S, Fuchs H, Paton V, Bajamonde A (2001). Use of chemotherapy plus a monoclonal antibody against HER2 for metastatic breast cancer that overexpresses HER2. N Engl J Med.

[11] Marty M, Cognetti F, Maraninchi D, Snyder R, Mauriac L, Tubiana-Hulin M (2005). Randomized phase II trial of the efficacy and safety of trastuzumab combined with docetaxel in patients with human epidermal growth factor receptor 2-positive metastatic breast cancer administered as first-line treatment: the M77001 study group. J Clin Oncol.

[12] Rugo H, Brammer M, Zhang F, Lalla D (2010). Effect of trastuzumab on health-related quality of life in patients with HER2-positive metastatic breast cancer: data from three clinical trials. Clin Breast Cancer.

[13] McIntyre K, O'Shaughnessy J, Schwartzberg L, Gluck S, Berrak E, Song JX (2014). Phase 2 study of eribulin mesylate as first-line therapy for locally recurrent or metastatic human epidermal growth factor receptor 2-negative breast cancer. Breast Cancer Res Treat.

[14] Wilks S, Puhalla S, O'Shaughnessy J, Schwartzberg L, Berrak E, Song J (2014). Phase 2, multicenter, single-arm study of eribulin mesylate plus trastuzumab as first-line therapy for locally recurrent or metastatic HER2-positive breast cancer. Clin Breast Cancer..

[15] Osoba D, Rodrigues G, Myles J, Zee B, Pater J (1998). Interpreting the significance of changes in health-related quality-of-life scores. J Clin Oncol.

[16] Cocks K, King MT, Velikova G, Martyn St-James M, Fayers PM, Brown JM (2011). Evidence-based guidelines for determination of sample size and interpretation of the European organisation for the research and treatment of Cancer quality of life questionnaire Core 30. J Clin Oncol.

[17] Efficace F, Biganzoli L, Piccart M, Coens C, Van Steen K, Cufer T (2004). Baseline health-related quality-of-life data as prognostic factors in a phase III multicentre study of women with metastatic breast cancer. Eur J Cancer.

[18] Kramer JA, Curran D, Piccart M, de Haes JCJM, Bruning P, Klijn J (2000). Identification and interpretation of clinical and quality of life prognostic factors for survival and response to treatment in first-line chemotherapy in advanced breast cancer. Eur J Cancer.

[19] Quinten C, Martinelli F, Coens C, Sprangers MA, Ringash J, Gotay C (2014). A global analysis of multitrial data investigating quality of life and symptoms as prognostic factors for survival in different tumor sites. Cancer..

[20] Svensson H, Hatschek T, Johansson H, Einbeigi Z, Brandberg Y (2012). Health-related quality of life as prognostic factor for response, progression-free survival, and survival in women with metastatic breast cancer. Med Oncol.

[21] Carrick S, Parker S, Wilcken N, Ghersi D, Marzo M, Simes J. Single agent versus combination chemotherapy for metastatic breast cancer. Cochrane Database Syst Rev. 2005:CD003372. 10.1002/14651858.CD003372.pub2 15846660

[22] Heidemann E, Stoeger H, Souchon R, Hirschmann WD, Bodenstein H, Oberhoff C (2002). Is first-line single-agent mitoxantrone in the treatment of high-risk metastatic breast cancer patients as effective as combination chemotherapy? No difference in survival but higher quality of life were found in a multicenter randomized trial. Ann Oncol.

[23] Joensuu H, Holli K, Heikkinen M, Suonio E, Aro AR, Hietanen P (1998). Combination chemotherapy versus single-agent therapy as first- and second-line treatment in metastatic breast cancer: a prospective randomized trial. J Clin Oncol.

[24] Brunner KW, Cavalli F (1987). Evaluation criteria in comparative clinical trials in advanced breast cancer: a proposal for improvement. Endocrine therapy of breast cancer II: current developments and new methodologies.

[25] de Haes JCJM, van Knippenberg FCE, Neijt JP (1990). Measuring psychological and physical distress in cancer patients: structure and application of the Rotterdam symptom checklist. Br J Cancer.

[26] Nabholtz JM, Senn HJ, Bezwoda WR, Melnychuk D, Deschenes L, Douma J (1999). Prospective randomized trial of docetaxel versus mitomycin plus vinblastine in patients with metastatic breast cancer progressing despite previous anthracycline-containing chemotherapy. 304 study group. J Clin Oncol.

[27] Simes RJ, Gebski VJ, Coates AS, Forbes J, Harvey V, Van Hazel G (1994). Quality of life (QOL) with single agent mitozantrone (MTZ) or combination chemotherapy (CMFP) for advanced breast cancer: a randomised trial [abstract]. Proc Am Soc Clin Oncol.

[28] Spitzer WO, Dobson AJ, Hall J, Chesterman E, Levi J, Shepherd R (1981). Measuring the quality of life of cancer patients: a concise QL-index for use by physicians. J Chronic Dis.

[29] Dear RF, Mc Geechan K, Jenkins MC, Barratt A, Tattersall MHN, Wilcken N (2013). Combination versus sequential single agent chemotherapy for metastatic breast cancer. Cochrane Database Syst Rev.

[30] Osoba D, Slamon DJ, Burchmore M, Murphy M (2002). Effects on quality of life of combined trastuzumab and chemotherapy in women with metastatic breast cancer. J Clin Oncol.

[31] Cortes J, Baselga J, Im YH, Im SA, Pivot X, Ross G (2013). Health-related quality-of-life assessment in CLEOPATRA, a phase III study combining pertuzumab with trastuzumab and docetaxel in metastatic breast cancer. Ann Oncol.

[32] Gianni L, Romieu GH, Lichinitser M, Serrano SV, Mansutti M, Pivot X (2013). AVEREL: a randomized phase III trial evaluating bevacizumab in combination with docetaxel and trastuzumab as first-line therapy for HER2-positive locally recurrent/metastatic breast cancer. J Clin Oncol.

[33] Hurvitz SA, Dirix L, Kocsis J, Bianchi GV, Lu J, Vinholes J (2013). Phase II randomized study of trastuzumab emtansine versus trastuzumab plus docetaxel in patients with human epidermal growth factor receptor 2-positive metastatic breast cancer. J Clin Oncol.

