# Supplementary material for: Health-related quality of life in patients receiving first-line eribulin mesylate with or without trastuzumab for locally recurrent or metastatic breast cancer
Source: BMC Cancer. 2019 Jun 13;19:578. doi: 10.1186/s12885-019-5674-5 (PMC6567408; doi:10.1186/s12885-019-5674-5)
Supplement: Supplementary file 1 — Table S1. Summary of QLQ-C30 and QLQ-BR23 Baseline Scores. (DOCX 16 kb) [file 12885_2019_5674_MOESM1_ESM.docx]

**Supplementary Table 1**. Summary of QLQ-C30 and QLQ-BR23 Baseline Scores

| **QLQ-C30** | | | | |
| --- | --- | --- | --- | --- |
|  | **Study 206** | | **Study 208** | |
| **Symptoms/Scales** | **n** | **mean (SD)** | **n** | **mean (SD)** |
| GHS/QoL | 56 | 64.7 (23.1) | 51 | 65.5 (25.1) |
| Physical functioning | 56 | 75.0 (24.5) | 51 | 76.3 (26.6) |
| Role functioning | 56 | 64.9 (36.5) | 51 | 74.8 (35.3) |
| Emotional functioning | 56 | 70.5 (23.1) | 51 | 67.3 (29.1) |
| Cognitive functioning | 56 | 85.4 (22.7) | 51 | 85.3 (18.2) |
| Social functioning | 56 | 70.2 (31.3) | 51 | 76.8 (32.2) |
| Fatigue | 56 | 41.9 (29.4) | 51 | 32.0 (30.0) |
| Nausea and vomiting | 56 | 10.7 (17.2) | 51 | 7.5 (18.4) |
| Pain | 56 | 37.2 (30.5) | 51 | 32.7 (34.3) |
| Dyspnea | 56 | 30.9 (34.7) | 51 | 24.8 (30.4) |
| Insomnia | 56 | 39.3 (34.3) | 51 | 35.3 (31.6) |
| Appetite loss | 56 | 27.4 (28.5) | 51 | 22.2 (29.6) |
| Constipation | 56 | 19.6 (22.7) | 51 | 19.6 (26.0) |
| Diarrhea | 56 | 8.9 (21.6) | 51 | 7.2 (19.2) |
| Financial difficulties | 56 | 29.2 (33.1) | 51 | 32.0 (34.0) |
| **QLC-BR23** | | | | |
|  | **Study 206** | | **Study 208** | |
| **Symptoms/Scales** | **n** | **mean (SD)** | **n** | **mean (SD)** |
| Body image | 56 | 80.4 (26.8) | 51 | 78.6 (26.7) |
| Sexual functioning | 54 | 12.4 (17.2) | 47 | 16.3 (24.2) |
| Sexual enjoyment | 10 | 43.3 (27.5) | 15 | 64.5 (26.6) |
| Future perspective | 53 | 44.0 (33.8) | 51 | 41.2 (35.7) |
| Systemic therapy SEs | 56 | 17.1 (13.6) | 51 | 15.5 (14.8) |
| Breast symptoms | 54 | 15.0 (24.8) | 51 | 22.4 (26.6) |
| Arm symptoms | 56 | 19.8 (23.9) | 51 | 22.2 (23.7) |
| Upset by hair loss | 6 | 22.2 (40.4) | 3 | 22.2 (19.2) |

GHS, global health status; QoL, quality of lif
